# Pragmatischere randomisierte Studien mit Fokus auf Registerbasierung

**DOI:** 10.1007/s11553-022-00974-w

**Published:** 2022-08-22

**Authors:** Stefan Lange, Jörg Lauterberg

**Affiliations:** Institut für Qualität und Wirtschaftlichkeit im Gesundheitswesen – IQWiG, Im Mediapark 8, 50670 Köln, Deutschland

**Keywords:** Versorgungsnahe Datenerhebung, Registerbasierter RCT, Externe Validität, Generalisierbarkeit, PRECIS‑2, Routine practice data collection, Registry-based randomized controlled trials, External validity, Applicability, PRECIS‑2

## Abstract

**Hintergrund:**

Vom Studiendesign her stark einschränkende randomisiert kontrollierte Studien (RCT) mit hoch selektierten Teilnehmern und Bedingungen liefern Ergebnisse, deren Übertragbarkeit auf die klinische Routineversorgung und Nützlichkeit für Erstattungsentscheidungen bisweilen bezweifelt wird.

**Fragestellung:**

Bieten vor dem erwähnten Hintergrund pragmatisch orientierte RCT und registerbasierte RCT Lösungspotenziale? Welche Chancen und Risiken sind mit pragmatischeren Studien verbunden, und welche methodischen Aspekte sind besonders zu beachten?

**Methoden:**

Der Beitrag zeigt eine narrative Übersicht zu pragmatisch orientierten RCT und registerbasierten RCT mit Vorstellung des PRECIS-2-Ansatzes („pragmatic-explanatory continuum indicator summary“) sowie einer Darstellung von Beispielstudien mit Diskussion methodischer Aspekte.

**Ergebnisse:**

Klinische RCT zur vergleichenden Nutzenbewertung sind auf einem Kontinuum zwischen den Polen „sehr pragmatisch“ und „sehr explanatorisch“ angesiedelt. Eine Grenze, ab der ein RCT als pragmatisch bezeichnet wird, ist nicht konsentiert. Pragmatischere RCT sind häufig gekennzeichnet durch wenig selektierte, aber dafür große Patientengruppen, Einbettung in ein Normalversorgungssetting und patientenrelevante Outcomes. Sie verzichten meist auf nachhaltige Adhärenzsicherung der initial zugeordneten Behandlung, auf Verblindung und aufwendige Zwischenuntersuchungen. Dies kann allerdings zu interpretatorischen Problemen führen, v. a. wenn sich keine Interventionsunterschiede zeigen.

**Schlussfolgerungen:**

Pragmatischere randomisierte Studien und registerbasierte RCT haben das Potenzial, mit ihren Ergebnissen zu wichtigen Entscheidungsgrundlagen für die klinische Praxis, aber auch für die Gesundheitspolitik und Erstattungsfragen zu werden. Um dieses Potenzial zu heben, sind allerdings noch manche Hürden vor allem gesetzlicher Art zu beseitigen.

## Abstract (en)

**Background:**

Randomized controlled trials (RCTs) that are highly restrictive in terms of study design, with highly selected participants and conditions, produce results whose applicability to routine clinical care and usefulness for reimbursement decisions is sometimes questioned.

**Objectives:**

In view of the background mentioned above, do more pragmatic RCTs and registry-based RCTs offer potential solutions? What are the opportunities and risks associated with more pragmatic studies and which methodological aspects should be given special attention?

**Methods:**

Narrative review of more pragmatic RCTs and registry-based RCTs with presentation of the Pragmatic Explanatory Continuum Indicator Summary 2 (PRECIS-2) approach, presentation of example studies, and discussion of methodological aspects.

**Results:**

Clinical RCTs for comparative effectiveness evaluation are located on a continuum from “very pragmatic” to “very explanatory”. No consented threshold exists from which an RCT is considered pragmatic. More pragmatic RCTs are often characterized by less selected but larger patient groups, embedding into a normal care setting, and patient-relevant outcomes. They usually dispense with longer-term assurance of patient adherence to the initially assigned treatment, blinding, and resource intensive intermediate examinations. However, this can lead to problems in interpretation, especially if no differences between interventions are shown.

**Conclusions:**

More pragmatic RCTs and registry-based RCTs have the potential to become an important basis for decision-making in clinical practice, but also for health policy and reimbursement issues. However, in order to realize this potential, a number of hurdles—especially legal ones—have to be abolished.

Randomisiert kontrollierte Studien (Randomized Controlled Trials – RCT), wie sie typischerweise für Zulassungsverfahren von Arzneimitteln durchgeführt werden, sind schon seit geraumer Zeit in die Kritik geraten. Sie seien in Planung, Genehmigung und Durchführung immer komplexer geworden, sehr teuer, dauerten lange, hätten nicht selten Rekrutierungsprobleme und es mangele ihnen an Repräsentativität. Denn ihre Ergebnisse würden u. a. wegen stark selektierter Studienpopulationen, der Durchführung in spezialisierten akademischen Zentren und wegen hochgradig kontrollierter Studienbedingungen als unzureichend übertragbar auf die klinische Routineversorgung gesehen [27].

Das limitiert aus Sicht der Protagonisten sog. „Real World Evidence“ ihren Erkenntnisnutzen für die Fundierung klinischer Behandlungsleitlinien ebenso wie für Erstattungsentscheidungen. Der Ruf nach zumindest ergänzender Berücksichtigung von Studien auf Grundlage versorgungsnaher Beobachtungsdaten (z. B. Daten von Krankenkassen, aus Patientenregistern oder aus elektronischen Patientenakten) ist daher für die genannten Zwecke, aber auch für den regulatorischen Kontext, immer vernehmbarer geworden [1].

Abgesehen davon, dass Beobachtungsstudien für Nutzenbewertungen gleichzeitig erheblich aufwendiger und anfällig sind für indikationsbezogenen Selektionsbias und Ergebnisverzerrungen durch unbekannte Störgrößen („confounder“), zeigen aktuelle Publikationen für den Arzneimittelbereich in ernüchternder Weise, dass zu regulatorischen Zwecken durchgeführte klinische RCT durch Analysen versorgungsnaher Daten nur in ganz geringer Zahl nachgebildet („emuliert“) werden können. So konnten Franklin et al. [8] in nur 9,5 % eines Pools von 589 betrachteten primären Zulassungsstudien und Wallach et al. [37] in keinem einzigen Fall von 50 konfirmatorischen Nachzulassungsstudien mit Krankenkassendaten die klinischen RCT nachbilden, weil in den Beständen entscheidende Daten zu Diagnosen/Indikationen, zur Interventionsdurchführung oder zu wichtigen Endpunkten fehlten.

Vor diesem Hintergrund könnte versorgungsnah und weniger eingeschränkt angelegten pragmatischeren randomisierten Studien eine besondere Bedeutung zukommen, da sie eine der Forschungsfragestellung jeweils angemessene Verbindung zwischen interner und externer Validität zu erreichen versuchen und zugleich mit weniger Aufwand durchführbar sind. Beispiel für den direkt in die Praxis implementierbaren Erkenntnisgewinn aus solchen Studien stellt die plattformbasierte RECOVERY-Studie [12] im Rahmen der Coronapandemie dar, die beispielsweise innerhalb kurzer Frist den Nutzen von Dexamethason in der Akuttherapie von COVID-19 („coronavirus disease 2019“) bei schwer Erkrankten zeigen konnte.

## Pragmatischere klinische Studien

Bereits 1967 beschrieben Schwartz und Lellouch [34] mit „explanatorischen“ und „pragmatischen“ Studien in einer Gegenüberstellung zwei Typen von Therapievergleichsstudien mit unterschiedlicher Zielsetzung. Erstere suchen durch hoch kontrollierte Designs und stark vorselektierte Populationen unerwünschte Ergebnisvariabilität zu reduzieren und so optimale Voraussetzungen für das valide Aufzeigen von Wirksamkeitsunterschieden zu schaffen („efficacy“). Letztere streben z. B. durch das Zulassen von Begleittherapien und Behandlungswechsel in möglichst wenig selektierten Patientengruppen an, verallgemeinerbare Entscheidungsunterstützung für die initiale Therapiewahl in der klinischen Alltagsversorgung zu geben („effectiveness“).

**Abb. 1:**
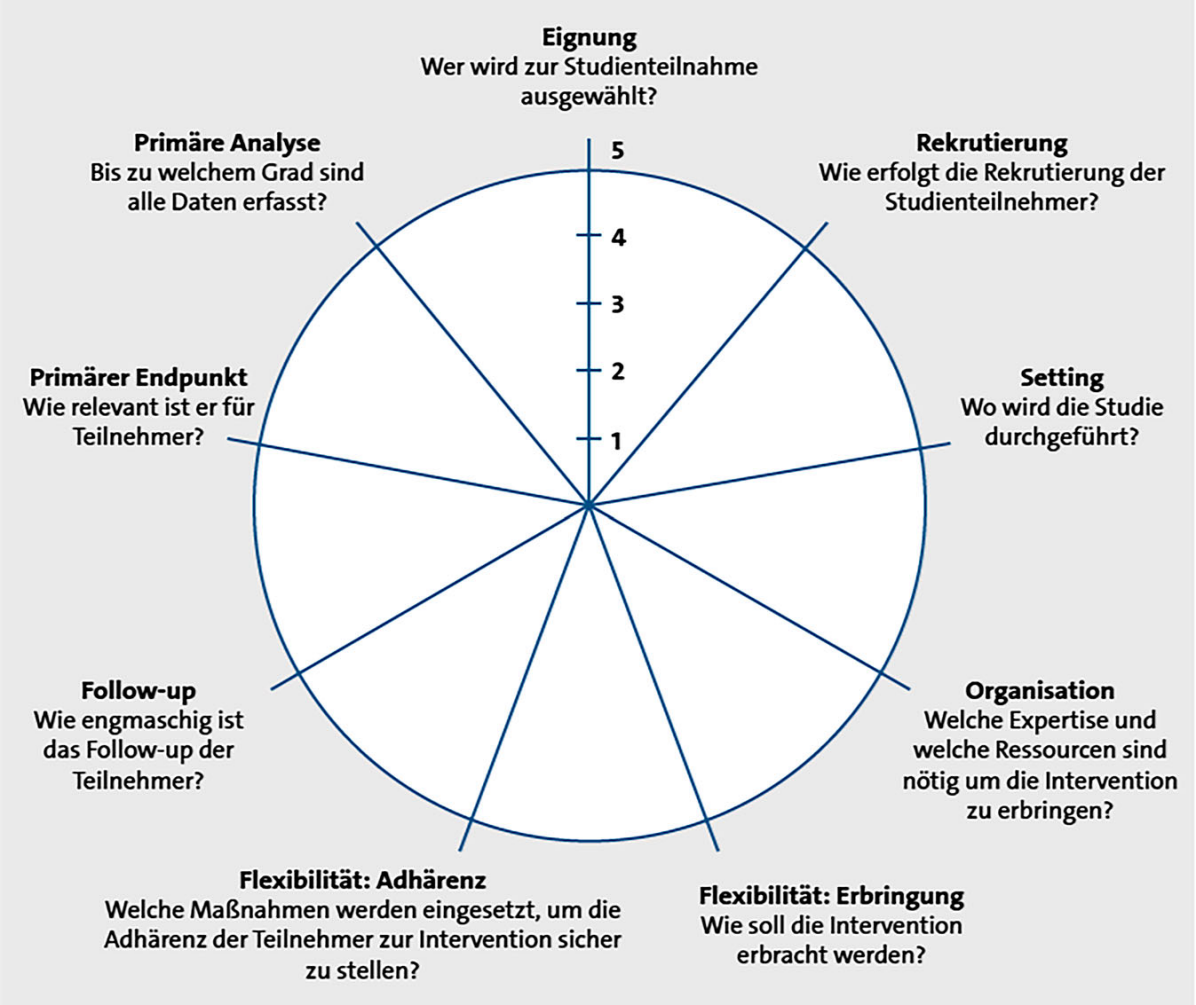
„Pragmatic-explanatory continuum indicator summary 2 (PRECIS-2) wheel“. (Mit freundl. Genehmigung entnommen und übersetzt aus: [22])

Inzwischen hat sich die Auffassung durchgesetzt, dass eine klinische Studie generell auf einem Kontinuum von „sehr explanatorisch“ bis „sehr pragmatisch“ angesiedelt ist, d. h. in ihrem Design im Mix sowohl pragmatische wie auch explanatorische Elemente enthalten kann [33]. Pragmatismus sollte als mehrdimensionales Konstrukt begriffen werden [6, 22]. Der Grad an Pragmatismus ergibt sich aus der Zusammenschau der im validierten PRECIS-2-Tool („pragmatic-explanatory continuum indicator summary“) betrachteten Studiendimensionen (Abb. 1 aus [22]).

Das Instrument wurde primär zur Unterstützung bei der Studiendesignplanung entwickelt, um strukturiert zu reflektieren, inwieweit die Studienbedingungen an die künftige klinische Routineversorgung angenähert sind. Es kann aber auch retrospektiv zur Studiencharakterisierung eingesetzt werden. Eine Gewichtung der Merkmalsdimensionen oder ein konkreter Schwellenwert, ab dem eine Studie insgesamt als pragmatisch bezeichnet werden kann [29], ist bislang nicht konsentiert worden. Allerdings halten Gamerman et al. [9] bei ihrem Versuch einer Begriffsbestimmung die 4 Designelemente versorgungsnahe Patientenpopulation, Einbettung in Normalversorgungssetting, angemessene Vergleichstherapie (aktiv) und versorgungs- bzw. patientenrelevante Outcomes für definitorische Schlüsselkriterien von Pragmatismus.

Die stärkere Berücksichtigung von pragmatischen Designelementen zielt auf eine Erhöhung der externen Validität und somit bessere Übertragbarkeit der Ergebnisse auf verschiedene Anwendungssituationen [40]. Allerdings ist die externe Validität einer Studie letztendlich kein exakt beschreibbares Merkmal, sondern muss in Bezug auf den jeweiligen Übertragungskontext idealerweise unter Kenntnis von relevanten Effektmodifikatoren situativ beurteilt werden. Dies gilt gemäß einem Grundpostulat der evidenzbasierten Medizin bezüglich der Übertragbarkeit externer Evidenz am Ende immer auch für die klinisch-therapeutische Einzelfallentscheidung in der Interaktion von Patient und Behandler(n).

Wenn Alter, Geschlecht, Multimorbidität, ethnische Herkunft der Patienten oder die Erfahrung der Therapeuten als Effektmodifikatoren einen bedeutsamen Einfluss auf die Therapieeffekte haben (könnten), dann ist es in pragmatisch orientierten Studien wichtig, diesbezügliche Selektion zu vermeiden und für einen breiten Einschluss von Teilnehmern zu sorgen. Die so erzeugte Heterogenität („Praxisrauschen“) und Ergebnisvariabilität sollte für Erkenntnisse zur Effektmodifikation (z. B. durch geeignete Subgruppenanalysen oder Bestimmung von Zentrumseffekten) weiter aufgeklärt werden, damit Wissen zur Generalisierbarkeit von Studienresultaten gewonnen werden kann. Voraussetzung für solche Analysen ist allerdings, dass alle relevanten Variablen etwa zur Subgruppencharakterisierung erhoben werden. Das bedeutet aber auch, dass eher pragmatisch angelegte Studien in der Regel höhere Fallzahlen aufweisen (müssen).

## PCORI-Ansatz

Wichtig ist die u. a. von der Food and Drug Administration (FDA; [35]) getroffene Feststellung, dass das explanatorische Element der Randomisierung der Teilnehmer auf die zu vergleichenden Interventionen kein grundsätzlicher Widerspruch zur versorgungsnahen Orientierung einer Studie ist. Bedeutsamer als die konkrete Studienmethodik ist den Autoren zufolge die Studiendurchführung in Settings mit wenig selektierten Teilnehmern, die der Routineversorgung entsprechen. Analog dazu hat das 2010 in den USA vom Kongress gesetzlich gegründete unabhängige Patient-Centered Outcomes Research Institute (PCORI) 4 Jahre später ein großes Forschungsdauerprogramm aufgesetzt, das mittels großer pragmatisch orientierter klinischer RCT Interventionen im Hinblick auf Nutzen und Schaden vergleichen soll [16]. Dabei handelt es sich um randomisierte Vergleiche von Arzneimitteln, nicht-medikamentösen Behandlungsverfahren, Präventionsmethoden und komplexen Versorgungsprogrammen. Die Forschungsfragestellungen adressieren den Wissens- und Entscheidungsbedarf von Patienten, versorgenden Angehörigen, Mitgliedern der Gesundheitsprofessionen sowie von Entscheidungs- und Leistungsträgern, welche über entsprechende Gremien auch an der Themenauswahl beteiligt sind. Intendiert ist eine möglichst rasche Implementierung der gewonnenen Erkenntnisse in die US-amerikanische Versorgungspraxis. Eine Auswahl geförderter Studien zeigt Tab. 1.

**Tab. 1:** PCORI-Förderung (Patient-Centered Outcomes Research Institute) von pragmatischen randomisierten klinischen Studien – Beispiele

Name (ggf. Webseite) der Studie und Registrierung	Fragestellung	Studienpopulation	Status	Beobachtungsdauer	Fördersumme MIO US $	Referenz
ADAPTABLE www.theaspirinstudy.org NCT02697916	Vergleich von 81 mg vs. 325 mg Aspirin täglich zur Prävention von Tod, Herzinfarkt und Schlaganfall sowie zur Minderung von Blutungsrisiken bei Patienten mit kardiovaskulärer Arteriosklerose	15.076 Patienten	Abgeschlossen	Median 26,2 Monate	19,1	[18]
PREPARE NCT02995733	Wirksamkeitsvergleich für 2 Asthmabehandlungsstrategien in erwachsenen Bevölkerungsgruppen mit hoher Krankheitslast bezgl. Asthmaanfällen, Notfallbehandlungen und Hospitalisierung	1.200 Patienten	Rekrutierung abgeschlossen	15 Monate	14,5	[2]
REGAIN NCT02507505	Vergleich von Regional- und Allgemeinanästhesie bei der Hüftgelenkfrakturchirurgie von über 50-Jährigen hinsichtlich der Gehfähigkeit nach 60 Tagen	1.600 Patienten	Abgeschlossen	60 Tage	11,8	[28]
CODA www.codastudy.org NCT02800785	Vergleich einer 10-tägigen Antibiose mit der Appendektomie bei erwachsenen Appendizitispatienten bezgl. der Lebensqualität nach 30 Tagen	1.552 Patienten	Abgeschlossen	Bei Studienende bis 4 Jahre Nachbeobachtung	13,2	[3]
Comparative Effectiveness of School-based Caries Prevention NCT03442309	Vergleich von 2 unterschiedlich aufwendigen halbjährlichen Kariesbehandlungs- und Kariesprophylaxeprogrammen bei Grundschulkindern aus einer ethnischen Minorität („hispanic/latino background“) mit geringem Einkommen	60 Grundschulen in New York	Rekrutierend	3–4 Jahre	13,3	[32]

Ein auf Pragmatismus zielendes Axiom der PCORI-Förderpolitik ist die klare Bevorzugung von patienten- und versorgungsrelevanten Zielgrößen vor Surrogatparametern wie etwa Laborwerten. Entsprechend werden in den Studien häufig Mortalität, Morbidität, Behinderungsgrad, Alltagskompetenz und Teilhabe, Lebensqualität sowie Hospitalisierung als Endpunkte betrachtet. Zur möglichst sicheren Aufdeckung von Interventionsunterschieden werden wegen der erwünschten Heterogenität entsprechend große Studienpopulationen über viele verschiedene Zentren eingeschlossen und oft länger als üblich nachbeobachtet. Um das Ziel der Generalisierbarkeit der Ergebnisse für die Routineversorgung zu erreichen, wird häufig auf explanatorische Studienelemente verzichtet. Dazu gehören etwa aufwändiges Training für teilnehmendes Personal, das ggf. unter Routinebedingungen gar nicht zu gewährleisten wäre, zusätzliche Datenerhebungen, engmaschige, u. U. artifizielle Kontrolluntersuchungen (z. B. mit Bildgebung zur Progressionsbestimmung in onkologischen Fragestellungen), Adhärenzsicherung durch dicht gestaffelte Patientenvisiten oder strikte und v. a. stark einschränkende Vorgaben für das klinische Management. Allerdings wird für robuste Interventionsvergleiche im Unterschied zum PRECIS-2-Ansatz [22] für „usual care“ als Komparator klar gefordert, dass auch diese Versorgung nach Maßgabe von klinischen Leitlinien als gerechtfertigt gelten kann und detailliert und in quantifizierbarer Form beschrieben ist. Andere Gruppen, z. B. das GetReal-Konsortium, heben jedoch klar hervor, dass die Verwendung von Behandlungsleitlinien zur Definition von „usual care“ zwar für die Standardisierung hilfreich sein kann, dies jedoch den Grad an Pragmatismus mindern und dadurch die Anwendbarkeit der Ergebnisse in der klinischen Routineversorgung einschränken kann [45]. Hinter der erwähnten PCORI-Forderung steht neben wissenschaftlichen Gründen auch die aus ethischer Perspektive berechtigte Absicht (z. B. durch Vorgaben im Studienprotokoll), eine qualitativ mangelhafte oder Substandardversorgung in der Vergleichsgruppe auszuschließen. Pragmatische Studien dürfen daher einem PCORI-Grundsatzpapier [30] zufolge keinem Laissez-faire-Ansatz folgen, sondern erfordern eine sorgfältige, zielgerichtete Planung, um interne und externe Validität zu optimieren.

## Methodische Probleme von pragmatisch orientierten randomisierten klinischen Studien

Weil in fast allen pragmatischen orientierten RCT auf aufwendige Maßnahmen (z. B. engmaschige Kontrollvisiten) verzichtet wird, Patienten zur Beibehaltung der anfangs zugelosten Therapieform zu motivieren (Adhärenzsicherung), kann es durch Behandlungswechsel der Patienten zur Angleichung der Studienarmergebnisse kommen. Dies geschieht mitunter in einem solchen Ausmaß, dass das bewährte Intention-to-treat-Auswertungsprinzip (ITT) die Identifikation von Effektunterschieden erschwert. Dies ist insbesondere bei Nichtunterlegenheitsstudien problematisch, da hier eine Nivellierung das Studienziel begünstigen kann [6, 13]. So hatten in der ADAPTABLE-Studie [18] zur Aspirin-Prophylaxe bei kardiovaskulärer Arteriosklerose eine große Zahl von Patienten, die vor Studienbeginn die geringere Dosis von 81 mg pro Tag genommen hatten, dann aber in die 325 mg-Gruppe gelost wurden, im Laufe der Studienzeit wieder zur geringeren Dosis gewechselt. Die Studie konnte evtl. hierdurch bedingt trotz ihrer Größe statistisch keine Behandlungsunterschiede feststellen. Neben der Bedeutung, die in solchen Fällen der sorgfältigen Beschreibung und Aufklärung von Gründen der Non-Adhärenz zukommt, wird für pragmatische RCT die Validierung und Standardisierung von Auswertungsmethoden für erforderlich gehalten, die versuchen, beobachtete Effekte für Konfundierungs- und Selektionsbias nach der Randomisierung statistisch zu adjustieren [26]. Allerdings kann man diese nachträgliche „Bereinigung“ skeptisch betrachten, denn sie konterkariert in gewisser Weise die eigentliche Absicht, etwas über den Versorgungsalltag zu erfahren.

Viele pragmatisch orientierte Studien haben keine engmaschigen Nachuntersuchungsregime durch Forschungspersonal oder behandelnde Ärzte, sondern erheben im Follow-up patientenberichtete Endpunkte über schriftliche Fragebögen und Online-Surveys. Dies kann zu einem gravierenden Problem fehlender Daten führen, was auch die oft wichtige Erhebung von unerwünschten Ereignissen betrifft. Insoweit kommt digitalen Erhebungswerkzeugen wie von den Patienten leicht zu bedienenden Forschungs-Apps und einem stabilen Erhebungskontext wie bei in Patientenregistern eingebetteten Studien ein großes Optimierungspotenzial zur Vermeidung eines Loss-to-follow-up zu.

Verblindung als explanatorisches Element fehlt in den meisten pragmatischen Studien oder ist nur partiell für die Auswerter vorgesehen. Erwartungen von Patienten und Behandlern beeinflussen in der Routineversorgung die Ergebnisse, was in pragmatischen Studien daher nach Auffassung verschiedener Autoren [4, 36, 38] mitabgebildet und nicht durch Verblindung verhindert werden soll. Da insbesondere patientenberichtete Endpunkte (z. B. Symptome, Nebenwirkungen, gesundheitsbezogene Lebensqualität) durch Erwartungen beeinflusst werden, wird empfohlen, primär gut objektivierbare Endpunkte (z. B. Mortalität, Hospitalisierung, größere kardiovaskuläre Ereignisse) zu betrachten und mit den Ergebnissen bei den subjektiven Endpunkten abzugleichen [6, 17]. Zudem sollten zumindest diejenigen, die Endpunkte erheben, verblindet sein [38].

Mit dem multizentrischen Ansatz und den möglichst unselektierten Patientengruppen in pragmatischeren Studien verbindet sich häufiger die Notwendigkeit des Einschlusses größerer Teilnehmerzahlen, um bei der hierdurch erzeugten Heterogenität auch kleinere, aber relevante Effekte in einem Vergleich ausreichend sicher zeigen zu können. Dies scheint nur dann realistisch zu sein, wenn – wie etwa im PCOR-NET in den USA oder im National Health Service (NHS) in England – durch jahrelange nachhaltige Förderung und Anreizsetzung für landesweite Forschungsverbünde und -plattformen entsprechende Voraussetzungen für größere klinische Studien geschaffen wurden.

Ergebnisse eines PCORI-Workshops mit der kritischen Implementierungsanalyse von 3 geförderten Studien [29] zeigen, dass je nach Studienfragestellung und Setting in der Planung und für Designanpassungen während der Durchführung eine sorgfältige Balanceabwägung zwischen pragmatischen und explanatorischen Elementen erfolgen muss, um einen guten Kompromiss zwischen interner und externer Validität zu finden. In der REGAIN-Studie [28] zum Vergleich von Regional- und Allgemeinanästhesie bei der Hüftgelenkfrakturchirurgie (Tab. 1) war z. B. ein Problem, die Vorgaben für die Durchführung der Interventionen pragmatisch genug zu gestalten, um nicht zu viele Krankenhäuser von einer Teilnahme abzuhalten. Denn eine nicht unübliche zusätzliche intravenöse Sedierung zur Regionalanästhesie kann einen Übergang zur Allgemeinanästhesie bedeuten und den intendierten Vergleich beeinträchtigen. Der Kompromiss für das Protokoll bestand letztendlich darin, die Sedierung nur bis zum Erhalt einer Patientenreaktion auf Berührung oder Ansprache zu titrieren und den Sedierungsgrad einmalig während der Operation mit einer kurzen Standardskala zu dokumentieren. Wegen des häufiger notwendigen Verfahrensumstiegs bei Misslingen einer Spinalanästhesie und dem Problem, dann noch mit ausreichender Power tatsächliche Unterschiede statistisch nachweisen zu können, folgte man präspezifiziert den Empfehlungen von Hernán und Robins [13], die geplante ITT-Auswertung durch eine Per-Protokoll-Analyse zu ergänzen. Außerdem wurden Kliniken mit auffällig hoher Cross-over-Rate gezielt vom Studienpersonal kontaktiert, um Verbesserungsmöglichkeiten zu besprechen und mit vertretbarem Aufwand die Adhärenz zu steigern.

Abschließend sei auf eine Artikelserie verwiesen, die sich als Ergebnis des von der EU geförderten „GetReal-Projekts“ detaillierter als hier möglich mit methodischen Herausforderungen von pragmatischen Studien beschäftigt [44].

## Registerbasierte randomisiert kontrollierte Studien (RRCT)

Frei übersetzt definieren die Autoren des ersten Standardhandbuchs dazu [10] ein Patientenregister als ein organisiertes System, das mit der Methodik einer Beobachtungsstudie einheitliche Daten (klinische und andere) sammelt, die bei definierten Populationen von Erkrankten, Exponierten oder Merkmalsträgern der Evaluation festgelegter Endpunkte dienen und das vorab bestimmte wissenschaftliche, klinische oder programmatische Zwecke verfolgt.

Die RCT, die sich in ihrer Durchführung auf vorhandene Patientenregister stützen, werden wegen der damit vermuteten Erhöhung externer Validität und der Reduktion von Kosten und administrativem Aufwand als Studientyp mit hohem Potenzial angesehen [20]. Sie werden von Li et al. [21] wie folgt definiert:

Registerbasierte randomisiert kontrollierte Studien sind pragmatische Studien, die Register als Plattform für eine oder mehrere Studienaktivitäten nutzen einschließlich Patientenidentifikation, Randomisierung, Interventionsdurchführung, Follow-up und Outcomebewertung. (Übersetzung durch Autoren)

Unter der Voraussetzung einer hohen Qualität des Patientenregisters wie auch der randomisierten Studie werden als Vorteile von RRCT deren Möglichkeiten gesehen

- zur validen Bestimmung von Ursache-Wirkungs-Beziehungen zwischen Behandlung und Ergebnissen unter klinischen Routinebedingungen durch die RCT-Komponente,
- zu wichtigen forschungspraktischen Vereinfachungen (z. B. Infrastrukturnutzung, Rekrutierungsplanung, Datennutzung, Follow-up) und erheblichen Kostenersparnissen für die RCT,
- zur Identifikation von Selektionseffekten und analysebedürftiger Fragen durch die vergleichende Betrachtung der RCT-Population und der übrigen Registerpopulation,
- zur potenziell vollständigen Langzeitbeobachtung von Interventionseffekten und -risiken durch die RCT-Population sowie
- zur Identifikation klinisch relevanter Subgruppen in Bezug auf Nutzen und Schaden [41].

Leider muss man konstatieren, dass es bis heute weder für Datenbankrecherchen geeignete eindeutige Indexbegriffe für RRCT gibt noch einen definitorischen Konsens, welche Kriterien Studien erfüllen müssen, um unter diesem Terminus zusammengefasst zu werden [19]. Das erschwert sowohl die Erstellung vollständiger systematischer Übersichten wie auch methodologische Diskussionen zu RRCT.

Eine Übersicht zu 17 RRCT [19] mit Studieneinschluss basierend auf der Definition von Li et al. [21] zeigt, dass alle hier identifizierten Untersuchungen akademisch initiiert waren, in der Mehrheit multizentrisch unter Einschluss großer Patientenzahlen durchgeführt wurden und trotz längerer Nachbeobachtung nur minimale Verluste an Teilnehmern hatten. Die in diesem Scoping Review identifizierten klinischen Interventionsstudien mit kardiologischem Schwerpunkt waren fast alle 2‑armig mit klar definierten Endpunkten wie Mortalität und in bereits über Jahre konsolidierte Krankheits- oder Prozedurenregister eingebettet.

Eine weitere Übersichtsarbeit zu RRCT [24] hat mit einer breiteren Einschlussdefinition 71 RRCT identifiziert. Zielsetzung war eine Beschreibung von Studiencharakteristika und Designmerkmalen von RRCT, die hier definiert waren als Studien mit randomisierter Zuweisung verschiedener Interventionen an die Teilnehmer (Patienten, Krankenhäuser, Praxen) und entweder vorab oder erst später geplanter Nutzung von mindestens einem Outcome-Merkmal aus einem existierenden Patienten- oder nationalen Informationsregister mit Personenbezug. Ergebnis der Analyse war u. a., dass mehr als die Hälfte der Studien aus Dänemark und Schweden stammten, also Ländern ohne verbreitete Datenschutzsorgen der Bevölkerung in Hinsicht auf Erfassung und Nutzung von Patientendaten zu Forschungszwecken. Zu 45 % wurden Präventionsmaßnahmen wie Impfungen und Krebsscreenings evaluiert. In mehr als der Hälfte der RRCT wurde durch entsprechende Verknüpfungen (in 70 % über einen eindeutigen Personenidentifikator) mehr als ein Register genutzt, wobei der primäre Endpunkt der Studie in fast 82 % der Fälle über ein Register bestimmt wurde. Fast 90 % der RRCT waren 2‑armig, in 6 Fällen verblindet und meist nur mit einer geringen Drop-out-Rate belastet. Die Autoren bedauern, dass sich nur für 8 der 71 RRCT in den Publikationen Angaben zur Qualität der Registerdaten fanden.

Mc Cord et al. [25] stellen in einem Vergleich von üblichen RCT mit solchen, die auch in der Versorgungsroutine gesammelte Daten miteinbeziehen, fest, dass über 30 auf Patientenregisterauswertungen beruhende Studien für 14 klinische Fragen mit 0,86 („ratio of odds ratios“) geringere Effekte für die betrachteten Interventionen zeigten.

Die Tab. 2 führt einige Beispiele von RRCT auf, die erkennbar Fragestellungen adressieren, die aus Public-health-Sicht große Bedeutung haben und/oder von hoher Relevanz für Patienten oder Behandler in der klinischen Praxis sind.

**Tab. 2:** Beispiele für registerbasierte randomisiert kontrollierte Studien (RRCT)

Name der Studie und Registrierung	Fragestellung	Studienpopulation	Register	Status	Beobachtungsdauer	Referenz
SPIRRIT-HFPEF NCT02901184	Vergleich von „usual care“ und Spironolacton mit „usual care“ bei Herzinsuffizienzpatienten mit erhaltener Auswurffraktion bezgl. kardiovaskulärer Todesrate und herzinsuffizienzbedingten Hospitalisierungen	3.200	Herzinsuffizienzregister Schweden verknüpft mit weiteren nationalen Registern	Rekrutierung	Bis zu 5 Jahren nach Studienbeginn	[23]
How to Best Treat Anterior Cruciate Ligament Injuries NCT04770233	Bei Patienten mit vorderer Kreuzbandruptur wird eine sofortige Operation mit anschließender Physiotherapie verglichen mit primärer Physiotherapie und – falls indiziert – späterer Operation, mit Bezug zur patientenberichteten Kniefunktion	328	Norwegisches Nationales Kniebänder-Register	Rekrutierung	10 Jahre	[5]
PyloResPes DRKS00018842	Vergleich von Pylorusresektion mit Pyloruserhalt bei der Pankreatoduodenektomie in Hinsicht auf die Häufigkeit postoperativer Magenentleerungsstörungen	984	StuDoQ/Pankreasregister Deutsche Gesellschaft für Allgemein- und Viszeralchirurgie	Rekrutierung	30 Tage	[31]
Heavy weight versus medium weight mesh in ventral hernia repair NCT03082391	Auswirkung des Netzgewichts bei der offenen retromuskulären Reparatur einer Bauchhernie auf die Schmerzsymptomatik ein Jahr nach der Operation	352	Register der Digestive Disease and Surgery Institute Quality Collaborative (DDSI-QC) und der Americas Hernia Society Quality Collaborative (AHSQC)	Abgeschlossen	1 Jahr	[43]
DETO2X-AMI NCT01787110	Vergleich einer initialen Sauerstoffgabe (6–12 h) mit Umgebungsluft für Patienten mit Herzinfarktverdacht und normaler Sauerstoffsättigung bezogen auf die generelle Einjahresmortalität	6.629	Nationales SWEDEHEART-Register Schweden	Abgeschlossen	1 Jahr	[14]

## Methodische Probleme und Herausforderungen bei RRCT

**Abb. 2:**
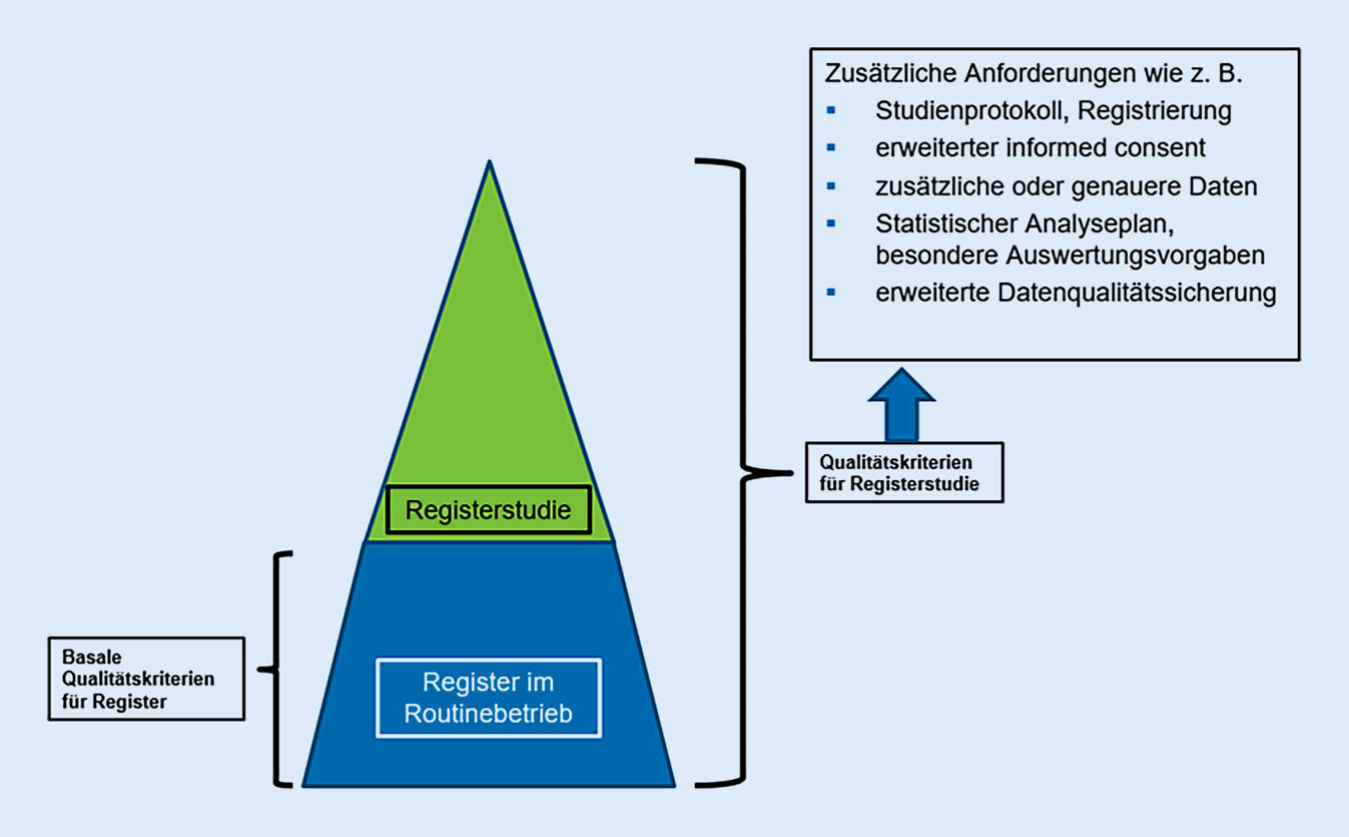
Qualitätskriterien von Patientenregistern und Registerstudien

Allgemein treffen für RRCT die für pragmatischer orientierte RCT zuvor angesprochenen methodischen Herausforderungen ebenfalls zu. Nach Li et al. [21] hängen typische Probleme von RRCT nicht mit der RCT-Methodik zusammen, sondern vorrangig mit den Registereigenschaften, die die Qualitätsbasis für die Durchführung registerbasierter Studien darstellen und die ausführlich in einem Rapid Report des IQWiG zu versorgungsnahen Datenerhebungen thematisiert wurden (Abb. 2; [15]).

Bei der Abwägung, ob ein vorhandenes Patientenregister für die Einbettung eines RCT geeignet sein könnte, kommt der Datenqualität von Basisvariablen und Outcomes entscheidende Bedeutung zu, die in der Abbildung dem Sockel des Dreiecks zugeordnet ist. Zentral sind hier die Kriterien Vollständigkeit, Vollzähligkeit und Korrektheit der routinehaft im Register erhobenen Daten. Zusätzlich kommen der Spitze des Dreiecks zugeordnet und im Kasten beispielhaft beschrieben bei einem RRCT weitere Qualitätsanforderungen zum Tragen, die für die Studie erfüllt sein müssen.

Fragen, die bei RRCT eine Rolle spielen, sind folgende: Wie viele zusätzliche Maßnahmen zur Qualitätssicherung der Datenerhebung (z. B. externe Audits, Quelldatenverifizierung [„data source verification“], Schulungen, Endpunktadjudizierung) müssten zur Erhöhung der internen Validität im Einzelfall ergriffen werden? Enthält der Registerdatensatz bereits alle wichtigen Variablen in Hinsicht auf die Fragestellung oder müssen viele zusätzliche Daten erhoben werden, was die Akzeptanz bei den Datenerfassern in den Zentren einschränken könnte? Hat das Register ein effektives Management und eine stabile Finanzierung, und ist es z. B. durch ein in die IT-Lösung integriertes Randomisierungsmodul technisch gut auf eine RCT-Durchführung vorbereitet? Inwieweit ist das Verfahren des Registers zur informierten Einwilligung der Patienten an die Erfordernisse eines RCT anpassbar und ergeben sich neben Datenschutzproblemen gegebenenfalls auch ethische, z. B. bei Studien zu (lebensbedrohlichen) Akuterkrankungen, wo der zeitnahe Patienteneinschluss eine große Rolle spielt [19]? Neben den Herausforderungen, die mit diesen Fragen bzw. deren Beantwortung verbunden sind, ist die mangelnde Verfügbarkeit von Patientenregistern mit nachweisbar hoher Qualität allerdings derzeit die größte Hürde für eine breite Durchführbarkeit von RRCT [20].

## Ausblick

Pragmatischere randomisierte Studien und RRCT haben das Potenzial, mit ihren Ergebnissen zu wichtigen Entscheidungsgrundlagen für die klinische Praxis, aber auch für die Gesundheitspolitik und Erstattungsfragen zu werden. Risiken für die Generierung robuster Evidenz und guter Übertragbarkeit auf die normale Gesundheitsversorgung ergeben sich aber aus den diskutierten methodischen Herausforderungen. Das heißt für das Gelingen pragmatischerer RCT konkret, dass eine der Fragestellung und dem intendierten Anwendungskontext angemessene Balance zwischen explanatorischen und pragmatischen Designelementen erreicht wird und somit interne und externe Validität gut verbunden sind. Eine Maximierung von Pragmatismus in einer klinischen Studie mit der Folge gravierender Verluste an interner Validität bedeutet immer auch den Verlust an externer Validität, denn grob verzerrte Studienergebnisse sind auf keinen Kontext übertragbar.

Die RRCT stellen als Studientyp generell, aber auch im Bereich der Onkologie [7] und selbst für seltene Erkrankungen [11] eine aussichtsreiche Option dar, zumal die meisten Patientenregister mit der Zielsetzung der Sicherung von Versorgungsqualität geschaffen wurden und insofern die angesprochene Gefahr von Substandardversorgung unter Usual-care-Bedingungen in den Vergleichsgruppen vermutlich geringer ist. Allerdings sind für das Heben dieser Potenziale in Deutschland zwei Voraussetzungen für die klinische Forschung essentiell: Erstens bedarf es wie in den USA, Skandinavien oder England eines klaren politischen Willens, diese Art von bislang überwiegend akademisch getriebener Forschung zur Beantwortung wichtiger klinischer Fragen aus Sicht von Patienten, Behandlern, Kostenträgern und weiteren Entscheidern im Gesundheitswesen zu fördern. Dazu gehören, wie vom Wissenschaftsrat 2018 empfohlen [42], neben der Einzelprojektförderung die Finanzierung und Etablierung verlässlicher Forschungsinfrastrukturen wie von Clinical Trial Units in Kliniken und dauerhaften, breit aufgestellten Forschungsverbünden im stationären und ambulanten Bereich sowie der Ausbau von qualitativ vielversprechenden Patientenregistern zu Forschungsplattformen mit der Kompetenz zur Durchführung von RRCT. Zweitens stellen die gesetzlichen Anforderungen für RRCT mit zugelassenen Therapien, die (nur) wegen der Randomisierung im Unterschied zu Beobachtungsstudien oder unsystematisch entstandenen Fallserien mit Erprobung selbst neuer Interventionen gestellt werden, für diesen vielversprechenden Ansatz versorgungsnaher Forschung eine erhebliche Hürde und fragwürdige Überregulierung dar [39].

Vor dem Hintergrund des Potenzials von pragmatischeren randomisierten Studien einschließlich von RRCT muss mit Blick auf die Ausgestaltung der anwendungsbegleitenden Datenerhebungen nach § 35a Abs. 3b SGB V für unzureichend erforschte neue Arzneimittel auch der Ausschluss randomisierter Vergleiche als nicht sinnvoll erachtet werden. Warum sollen die in der Aussagensicherheit eingeschränkten, komplexen und mit hohem Analyseaufwand [15] verbundenen Möglichkeiten, nach der Marktzulassung mit Beobachtungsdaten Nutzenvergleiche zu erstellen, nicht zumindest ergänzt werden durch weniger aufwendige (registerbasierte) randomisierte Phase-IV-Studien?

## Fazit für die Praxis

- Zu den Hauptmerkmalen pragmatischerer Studien gehören möglichst wenig restriktive Ein- und Ausschlusskriterien (Patienten, Einrichtungen) und die Berücksichtigung patientenrelevanter Outcomes.
- Mit dem PRECIS-2-Tool („pragmatic-explanatory continuum indicator summary“) lässt sich in der Studienplanung der notwendige Mix von pragmatischen und explanatorischen Designelementen systematisch reflektieren.
- Registerbasierte randomisiert kontrollierte Studien (RRCT) haben bei hoher Qualität des Patientenregisters das Potenzial, mit geringerem Aufwand in guter Verbindung von interner und externer Validität Fragen zum klinischen Nutzen von Interventionen zu beantworten.
- Pragmatischere randomisiert kontrollierte Studien (RCT) zur Nutzenbewertung in Deutschland bedürfen verstärkter Förderung mit einer Entwicklung klinischer Forschungsplattformen unter Vernetzung von Einrichtungen der Routineversorgung.
- Regulatorische Hindernisse zur Durchführung von registerbasierten RCT und Plattformstudien müssen abgebaut werden.
